# Respiratory Failure Following Organophosphate Poisoning: A Literature Review

**DOI:** 10.7759/cureus.1651

**Published:** 2017-09-03

**Authors:** Pirthvi Raj Giyanwani, Ujala Zubair, Osama Salam, Zarafshan Zubair

**Affiliations:** 1 Civil Hospital Karachi, Dow University of Health Sciences (DUHS), Karachi, Pakistan; 2 Medicine, Dow University of Health Sciences (DUHS), Karachi, Pakistan; 3 Sindh Medical College, Dow University of Health Sciences (DUHS), Karachi, Pakistan; 4 MBBS, Dow University of Health Sciences (DUHS), Karachi, Pakistan

**Keywords:** organophosphate poisoning, respiratory failure, clinical trials

## Abstract

Organophosphates (OPs) account for a large portion of suicides globally. OP manifests as cholinergic crises, which underlie respiratory failure. There are many pathways by which respiration is inhibited secondary to organophosphate poisoning. These include central as well as peripheral mechanisms, with central mechanisms predominating. We conducted a literature review in June 2017. PubMed, Embase, and Google Scholar were searched for studies that reported acute organophosphate poisoning in humans. In our review, data were collected from studies published during the years 2001 to 2016. The data consisted of 1,996 patients with organophosphate poisoning, of which 491 (24.6%) required ventilatory support secondary to respiratory failure. Treatment offered to OP poisoning patients should focus on its pathophysiology to benefit from the future outcomes. Recent advances direct the need for a central nervous system (CNS) protective strategy for future prevention and treatment of events associated with cholinergic crises.

## Introduction and background

Organophosphates (OPs) are one of the common causes of suicidality worldwide. Gunnell et al. have reported that there are 258,234 deaths each year from organophosphate poisoning, which accounts for about 30% of the suicidal cases globally [1]. OPs result in phosphorylation of serine hydroxyl residue on acetylcholine esterase enzyme, which results in the accumulation of acetylcholine. This leads to cholinergic features, which can be classified into central and peripheral. Peripheral events include vomiting, diarrhea, miosis, muscle fasciculations, urinary incontinence, and bronchoconstriction. Central effects include respiratory depression and delirium [2]. One of the devastating cholinergic features of organophosphate poisoning is respiratory failure. There are several explanations for respiratory failure; central, as well as peripheral mechanisms, underlie this phenomenon. However, studies have suggested that the major mechanisms regulating respiratory failure associated with OP ingestion are central in origin. The respiratory center known as the pre-Botzinger complex is situated in the ventrolateral medulla. It is composed of glutaminergic and muscarinic fibers. Excess acetylcholine can depress respiratory activity in these areas [3-4]. Injection of dichlorvos bilaterally into the pre-Botzinger complex in vagally intact rats produces a decrease in respiratory rate, a decrease in volume of inspired gas, and about 27% of the animals became apneic [3]. Similarly, when sarin nerve agent was injected bilaterally into the pre-Botziner complex, it produced respiratory arrest in rabbits by a rapid decrease in respiratory rate but without any change in tidal volume [5]. The sleep literature has been used to study the effects of acetylcholine on the brainstem, related to respiratory function. While an individual is in either natural or artificially induced rapid eye movement (REM) sleep, a surge of acetylcholine occurs in the pontomedullary reticular field of the brain stem, which inhibits respiration. Animal studies have shown the inhibition of respiration and a decrease in phrenic nerve output upon injection of acetylcholine in the exposed brainstem [6-7]. Vagus is the major neural pathway that interconnects the brain and lung. Mechanoreceptors provide feedback via the vagus nerve. Vagal mechanisms blunt the hypoventilation associated with OPs in spontaneously breathing animals. Vagal mechanisms also cause an increase in pulmonary secretions due to pulmonary irritants, and surgical vagotomy has been shown to decrease pulmonary secretions [8]. OP exposure to the lung causes increased acetylcholine at the pulmonary muscarinic receptors causing pulmonary abnormalities. OP agents increase the work of breathing through an increase in pulmonary static and dynamic compliance and by causing obstruction of airways [9-11]. OPs have the tendency to cause interstitial edema, which is responsible for the decrease in pulmonary compliance and ventilation-perfusion (V/Q) mismatch [12-13]. Another mechanism explaining the worsening of the respiratory function secondary to organophosphate poisoning is the induction of excitatory activity, which worsens cerebral hypoxia and compromises respiratory efforts [14]. Dickson et al. have concluded that the pretreatment of rats with diazepam has shown beneficial effects on survival in acute, severe OP poisoning. Diazepam prevents respiratory control overstimulation [14]. These effects of diazepam could be amplified if given along with peripherally acting anticholinergic agents that have no effect when given alone. Respiratory failure can be categorized into two forms depending on the time of onset from OPs ingestion. Early form is one that presents within 24 hours. Its pathophysiology can be explained by three mechanisms: depression of central respiratory drive from the respiratory center in the ventrolateral medulla, weakness of the muscles of respiration, and OP-induced bronchospasm and bronchorrhea induced via local and vagal mechanisms [15-17]. Explanations for late respiratory failure include peripheral dysfunction due to the sustained overstimulation of the neuromuscular junction (NMJ) [18-20]. Some researchers support the fact that late respiratory failure is due to direct OP toxicity of skeletal muscles, which causes the weakness and necrosis of skeletal muscle fibers. Peripheral acetylcholine at NMJ causes voluntary muscle weakness and fasciculations, including in the muscles of respiration [21].

## Review

Methodology

We conducted a literature review in June 2017. PubMed, Embase, and Google Scholar were searched for studies that reported acute organophosphate poisoning in humans. In our review, data were collected from studies published during the years 2001 to 2016 by using the keywords organophosphate poisoning, respiratory failure, and clinical trials. Data collection was restricted to studies in the English language only. The published data was included only. Inclusion criteria included studies reporting organophosphate poisoning (either intentional for suicidal purposes or accidental) in adults aged greater than 12 in any part of the world. The diagnosis of poisoning was clinically based on cholinergic signs and symptoms, however, pseudocholinesterase levels were done in few studies. All types of OPs were included in this study. The co-ingestion of OPs along with other agents was not included.

Results

A total number of eleven studies met our criteria and were included in this literature review. Data consisted of 1,996 patients with organophosphate poisoning, out of which 491 (24.6%) required ventilatory support secondary to the respiratory failure as mentioned in Table 1. Eddleston et al. have categorized their findings of respiratory failure into two categories: early onset and the late-onset. There were 90 patients who underwent respiratory failure. Out of those 90 patients, 61 patients (67.7%) had early onset respiratory failure, i.e., within 24 hours, including 52 patients who underwent respiratory failure within two hours of OP ingestion, whereas 29 patients (32.2%) had late onset respiratory failure. They observed in their study that patients having late respiratory failure required intubation for a longer duration versus those who were intubated within initial 24 hours (median time to first extubation 33 vs. 219 hrs, p<0.0001; median time to final extubation 45 vs. 284 hours, p<0.0001). Their study results report that patients requiring early intubation had their bronchorrhea and bronchospasm treated with atropine, however, there was no effect on Glasgow Coma Scale (GCS) or respiratory rate. Nineteen out of 25 patients (76%) who were poisoned with dimethoate and six out of the 21 patients (29%) who were poisoned with chlorpyrifos died [22]. Sungur et al. report an overall mortality rate of 27.6%, whereas the mortality rate was 50% among those who were on ventilatory support [23]. Indira et al. report an overall mortality of 28.4%, whereas the mortality rate was 40% among those with the intermediate syndrome [24]. Banday et al. have reported that the mortality was higher in patients who required ventilatory support for the duration of more than seven days (p<0.05) [25]. Two studies report tracheostomy done among patients. Hiremanth et al. report 21 patients (56.7%) out of 37 patients with tracheostomy, whereas Kang et al. report 17 patients (25%) out of 68 patients with tracheostomy [26-27]. 

**Table 1:** Estimates of respiratory failure following organophosphate poisoning

Study	Total No. of Patients	No. of Patients With Intermediate Syndrome	No. of Patients With Ventilatory Support	Mortality Rate (%)
Eddleston et al. - 2006 [22]	376	-	90	12.23%
Sungur et al. - 2001 [23]	47	-	10	27.6%
Indira et al. - 2013 [24]	176	31	26	28.4%
Banday et al. - 2015 [25]	133	-	53	33.3%
Hiremanth et al. - 2016 [26]	37	-	18	-
Kang et al. - 2009 [27]	68	-	35	19.1%
Jayawardane et al. - 2008 [28]	78	10	5	-
Panchal et al. - 2016 [29]	100	-	43	20%
Eddleston et al. - 2005 [30]	802	-	190	13.9%
Jayawardane et al. - 2008 [31]	78	-	12	-
Kumar et al. - 2014 [32]	101	-	35	9.9%
Total	1,996	-	491	

Discussion

Eddleston categorized his findings into early and late onset respiratory failure; however, he concluded that both of these syndromes overlap and it is difficult to classify patients into one of these categories. Patients experiencing an early form of respiratory failure had greater cholinergic signs and symptoms, whereas patients with delayed onset had fewer cholinergic signs and symptoms. Patients requiring intubation later than 24 hours required a longer duration of intubation and the longer duration of intubation is associated with greater mortality, as ventilator-associated factors contribute to morbidity and mortality. He also suggested that delayed respiratory failure can be due to the inappropriate administration of pralidoxime, as patients under his observation received only one day of intermittent pralidoxime therapy [22]. Samuel et al. suggested that the early administration of loading 1 g of pralidoxime could prevent late onset respiratory failure [33].

Hiremanth et al. formed two groups based on pseudocholinesterase (PChE) levels. Group A included patients with PChE levels greater than 1000 IU/L, whereas Group B included those having PChE levels less than 1000 IU/L. Group B patients had greater failure rates in weaning off mechanical ventilation, a higher rate of tracheostomy, a longer hospital stay, and a higher mortality rate [26]. APACHE II is used to measure the severity of disease in patients admitted to the intensive care unit. Kang et al. suggested that the APACHE II score was predictive of clinical outcomes [27]. The severity of clinical findings was also related to the kind of organophosphate ingested. The majority of studies do not categorize patients based on the type of OP ingested. Eddleston et al. reported that the worst clinical outcome was associated with dimethoate poisoning. The mainstay of treatment for organophosphate poisoning includes atropine and pralidoxime therapy. There are studies supporting the use of hemoperfusion for the management of severe OP poisoning; however, researchers are still investigating its therapeutic benefits. Burton et al. performed a study on rats; they found that atropine caused a depression of the phrenic nerve output and a depression of ventilatory output signals. Similarly, acetylcholine application was associated with an increase in ventilatory output signals and phrenic nerve output [34]. Atropine can switch off the production of secretions in pulmonary airways; however, it cannot help with the removal of secretions. Sympathetic stimulation is the mechanism that helps to remove the secretions. The therapy for OP poisoning is the same as it was 50 years back. Recent advances direct the need for the central nervous system (CNS) protective strategy for the future prevention and treatment of events associated with cholinergic crises. Studies have supported the fact that acute mortality via OP-induced respiratory failure is due to underlying central mechanisms rather than peripheral. No reduction in mortality rate was evidenced upon the administration of glycopyrrolate, which is a peripheral antagonist of acetylcholine. However, when it was given along with diazepam, the mortality rate was reduced. Gaspari et al. have observed apnea in animals with OP poisoning despite oxygenation and blood pressure regulation suggesting underlying central mechanisms [8,14].

Most patients die of complications secondary to ventilatory support, which include aspiration pneumonia and anoxic brain damage; however, in many clinical trials, these causes of death remain unreported.

## Conclusions

Respiratory failure following organophosphate ingestion carries very high mortality and morbidity rates. However, if pathophysiological mechanisms causing the respiratory failure are understood clearly, the future outcomes can be benefited either by preventing or by treating the respiratory failure effectively. We have explained, in our article, a few underlying mechanisms, including central mechanisms, for respiratory failure following organophosphate poisoning. However, further research is required in this field to direct CNS protective therapy for patients. The number of suicidal cases is increasing over the globe. Further research in this field can benefit the overall mortality outcome in patients with organophosphate poisoning.
